# Subcarrier Allocation Based Cooperative Spectrum Sharing with Wireless Energy Harvesting in OFDM Relaying Networks

**DOI:** 10.3390/s19122663

**Published:** 2019-06-13

**Authors:** Dan Huang, Mengshu Hou, Weidang Lu

**Affiliations:** 1 School of Computer Science and Engineering, University of Electronic Science and Technology of China, Chengdu 610054, China; huangdan@uestc.edu.cn; 2 College of Information Engineering, Zhejiang University of Technology, Hangzhou 310014, China; luweid@zjut.edu.cn

**Keywords:** cooperative spectrum sharing, power splitting, OFDM relaying, subcarrier allocation

## Abstract

In this paper, we propose subcarrier allocation based cooperative spectrum sharing protocol for OFDM relaying networks with wireless energy harvesting. In the proposed protocol, the cognitive relay node utilizes different subcarriers to forward the primary information to obtain the spectrum access for the cognitive system transmission. The primary system consists of two parts, a primary transmitter (PT) and primary receiver (PR), and cognitive system includes a cognitive source node (CSN), cognitive destination node (CDN) and cognitive relay node (CRN). In the first phase, CRN splits a fraction of the power received from the PT and CSN transmission to decode information, while the remaining power is used for energy harvesting. Then CRN uses disjoint subcarriers to forward the signals of PT and CSN by utilizing the harvested energy in the second phase. Three parameters which consist of power splitting ratio, power allocation and subcarriers allocation are optimized in our algorithm to maximize the cognitive transmission rate with the constraint of primary target transmission rate. Numerical and simulation results are shown to give useful insights into the proposed cooperative spectrum sharing protocol, and we also found that various system parameters have a great effect for the simulation results.

## 1. Introduction

Comparing with 3G and 4G networks, 5G network is able to support much higher data rate, which can provide service to not just smartphones but also majority of Internet of Things (IoT) devices. However, the spectrum utilized for the 5G network communication is limited, which will cause numerous IoT devices lack of spectrum to access to the 5G network.

To improve the spectrum utilization, cognitive radio (CR) allows the cognitive users (CUs) access to the licensed spectrum of the primary users (PUs) if it finds the spectrum wholly [1,2,3] or simultaneously transmit with PUs if the interference caused by CUs transmission is under the tolerant value [4]. Numerous works have been conducted to obtain reliable communications for CUs. Reference [5] proposed a sensing protocol to maximize cognitive network performance through learning the channel statistics. Reference [6] studied channel assignment for real-time traffic to obtain large capacity through elastic traffic assigning with small variance of capacity.

Cooperative relaying can enhance the communication performance through sharing antennas with neighboring users [7,8]. Combining cooperative relaying with cognitive radio will enable effective improvement of the spectrum sharing performance [9,10,11,12,13,14,15]. An opportunistic spectrum sharing protocol in OFDM relaying network is proposed in [9], in which the cognitive system gains spectrum access by amplifying and forwarding the primary signal. Reference [10] proposed OFDM two-way relaying cooperative spectrum sharing, in which the cognitive relay uses disjoint subcarriers to help forward the primary signals. Reference [13] analyzed performance of multi-relay collaborative spectrum sharing with incomplete CSI information, where cognitive users utilize collaborative zero-forcing beamforming. Through relaying the signal of the primary user, the cognitive user can obtain spectrum access. However, it needs to spend the power of cognitive user to forward the primary signal, which may cause the cognitive user to refuse to access the spectrum when it can only achieve poor performance.

Simultaneous wireless information and power transfer (SWIPT) technology has the ability to realize simultaneous information decoding and energy harvesting by receiving radio-frequency signals [16,17,18,19,20,21,22]. Integrating SWIPT with cooperative relaying spectrum sharing enables the cognitive relay to harvest the energy by receiving the primary signal simultaneously with information decoding, which makes the cognitive relay volunteer to forward the primary signal [23,24,25,26,27,28,29]. Reference [23] analyzes the outage probability of primary and cognitive users for two-way cognitive system based on SWIPT relaying, where the cognitive user uses the decode and forward (DF) protocol to help the primary users achieve bidirectional communication. Reference [25] studies the power allocation in NOMA relaying network, where the source uses a relay with the ability to harvest energy to forward for two users transmission. Reference [29] proposed cooperative spectrum sharing protocol in underlay cognitive relaying network, in which a SWIPT-enabled relay amplifies-and-forwards (AF) the information of the primary and cognitive users by using the harvested energy.

In the aforementioned existing SWIPT based cooperative spectrum sharing protocol, the primary and cognitive users interfere with each other, due to the fact that the cognitive relay uses the same spectrum to forward their signals. The interferences result in performance degradation for both the primary and cognitive users. To prevent the cognitive user from interfering with the primary user, in this paper, we propose a subcarrier allocation based cooperative spectrum sharing protocol with two transmission phases. The protocol requires OFDM relaying networks employing wireless energy harvesting, where the cognitive relay node utilizes different subcarriers to forward primary and cognitive signals. Specifically, in the first phase, the cognitive relay splits a fraction of the power received from the PT and CSN transmission to harvest energy. In order to implement SWIPT technology, we use the remaining power to decode information. In the second phase, the cognitive relay forwards PT and cognitive signals through utilizing disjoint subcarriers with the harvested energy in the first phase. We optimize the power splitting ratio, power allocation and subcarriers allocation for the cognitive transmission rate maximization problem with the constraint of primary target transmission rate.

The main contributions of this work are summarized as follows:

- Firstly, to prevent the interference between the primary and cognitive systems, we propose a subcarrier allocation based cooperative spectrum sharing protocol with wireless energy harvesting for OFDM relaying networks, in which the cognitive relay uses disjoint subcarriers to forward the signals of primary and cognitive systems.
- Secondly, we formulate a joint resource allocation scheme aiming to maximize the cognitive transmission rate through optimizing power splitting ratio, power allocation and subcarriers allocation with the constraint of primary target transmission rate.
- Finally, we provide numerical and simulation results to verify the proposed cooperative spectrum sharing protocol and find out the important effects of various system variables on the simulation results.

## 2. System Model and Protocol Description

**Figure 1:**
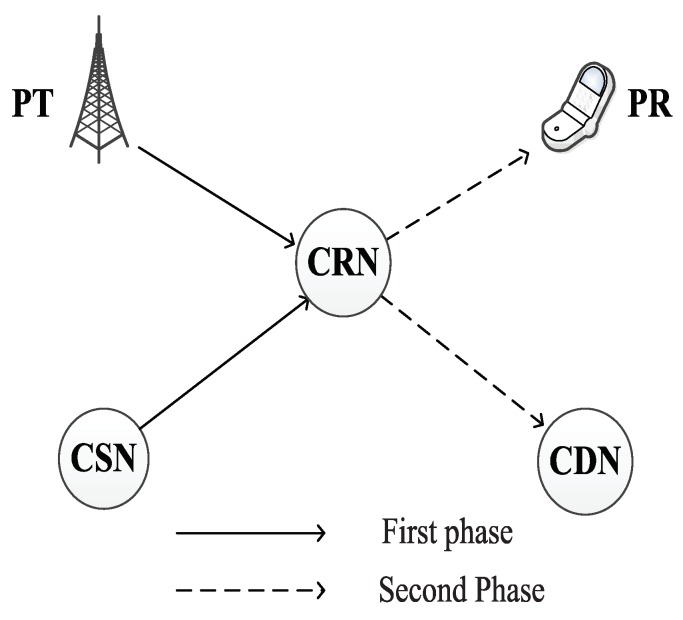
System model.

As shown in Figure 1, our proposed protocol consists of a primary system and a cognitive system. The primary system consists of a primary transmitter (PT) and primary receiver (PR), and the cognitive system comprises of a cognitive source node (CSN), cognitive destination node (CDN) and cognitive relay node (CRN). The primary system has the license to operate in a certain spectrum. We assume that PR keeps track of the signal-to-noise ratio (SNR) from PT to PR. The cognitive system can only opportunistically operate in this spectrum when the primary system is not able to achieve its target rate (e.g., the SNR from PT to PR is below a threshold due to path loss/shadowing/interference in a primary cellular network). It provides an opportunity for the cognitive system to access the primary spectrum. We use K={1,2,3,…,K} to denote the subcarriers set. We use $P_{p}$ and $P_{c}$ to denote the transmit power of PT and CSN, respectively.

In the proposed spectrum sharing protocol, the following handshake mechanism is adopted, inspired by the classic RTS (ready-to-send) and CTS (clear-to-send) signaling mechanism. When the SNR from PT to PR is below a threshold due to path loss, shadowing, or interference, resulting in the primary achievable rate falling below a target rate, PR will seek cooperation from neighboring terminals to enhance its transmission performance by sending out a request-to-cooperate (RTC) signal, in which the target rate is also embedded. This RTC signal is then responded to by PT with an acknowledge-to-cooperate (ATC) signal. Upon receiving both RTC and ATC signals, CRN is able to estimate the channel gains of PT→CRN and CRN→PR links, and calculate accordingly whether it is able to assist the primary system to achieve its target rate by serving as a cooperative DF relay. If positive, CRN responds by sending a confirm-to-cooperate (CTC) signal to PT and PR to indicate that it can cooperate with the primary system and the primary system correspondingly switches into a two-phase DF relaying mode, with CRN being the relay node. As a reward, the cognitive system can access the primary system to transmit its data. Otherwise, both primary system and cognitive system stop transmission.

## 3. Analysis of Achievable Rates

The primary system needs to predict its achievable rate based on the current SNR from PT to PR before deciding whether to send out the RTC signal. Without any cooperation from the cognitive system, the achievable rate of the primary system is given by

(1) $$R_{D}=\sum_{k\in K}\ln\left(1+p_{p,k}h_{PT-PR,k}\right)$$

where $h_{PT-PR,k}$ denotes the channel coefficients of PT→PR link on subcarrier *k*, $p_{p,k}$ denotes the transmit power over subcarrier *k* at PT following waterfilling approach.

When the achievable rate of the primary system $R_{D}$ falls below the target rate $R_{T}$, PR will seek cooperation from the neighboring nodes to improve its performance by sending out the RTC signal. This RTC is then responded to by PT with an ATC signal. We presume that the information regarding $P_{p}$ and $R_{T}$ is embedded in RTC and the channel state information of PT→PR link is embedded in ATC.

Next, the cognitive system decides, under the current channel condition, whether it is able to assist the primary system to reach $R_{T}$ by calculating the maximum instantaneous rate, $R_{max}$, of the primary system when CRN serves as a pure DF relay for the primary system [30]. If $R_{max}\geq R_{T}$, CRN will broadcast the CTC to both PR and PT to indicate that it can cooperate with the primary system, and the primary system correspondingly switches into a two-phase DF relaying mode, with CRN being the relay node. Otherwise, both the primary system and cognitive system stop transmission.

The two-phase DF relaying mode is operated through two equal time transmission phases. In the first phase, PT and CSN utilize all of the *K* subcarriers to transmit their signals to CRN. Thus, the signal received at CRN over subcarrier *k* can be denoted as

(2) $$y_{k}^{CRN}=\sqrt{p_{p,k}}x_{p,k}h_{PT-CRN,k}+\sqrt{p_{s,k}}x_{s,k}h_{CSN-CRN,k}+n_{1,k}$$

where $x_{p,k}$ and $x_{s,k}$ represent the primary and cognitive signal on subcarrier *k*, $h_{PT-CRN,k}$ and $h_{CSN-CRN,k}$ denote the channel coefficients of PT→CRN and CSN→CRN links on subcarrier *k*, $n_{1,k}∼\mathrm{CN}\left(0,\sigma_{1,k}^{2}\right)$ is the noise at CRN on subcarrier *k*, $p_{s,k}$ denotes the transmit power over subcarrier *k* at CSN following waterfilling approach.

CRN splits a fraction of received power with ratio λ to decode information, and another fraction with ratio (1−λ) to harvest energy. Then the harvested energy at CRN is given as

(3) $$E=\sum_{k\in K}\frac{1}{2}\left(1-\lambda \right)\eta \left(p_{p,k}{\left|h_{PT-CRN,k}\right|}^{2}+p_{s,k}{\left|h_{CSN-CRN,k}\right|}^{2}+\sigma_{1,k}^{2}\right)$$

where η denotes the energy conversion efficiency, which utilizes linear energy harvesting model [23,24,25,26,27,28,29].

The achievable rate of PT→CRN and CSN→CRN links in the first phase can be denoted as

(4) $$\begin{array}{ccc} R_{PT-CRN} & = & \sum_{k\in K}\frac{1}{2}\ln\left(1+\frac{\lambda p_{p,k}{\left|h_{PT-CRN,k}\right|}^{2}}{\lambda p_{s,k}{\left|h_{CSN-CRN,k}\right|}^{2}+\lambda \sigma_{1,k}^{2}+\sigma_{2,k}^{2}}\right) \end{array}$$

(5) $$\begin{array}{ccc} R_{CSN-CRN} & = & \sum_{k\in K}\frac{1}{2}\ln\left(1+\frac{\lambda p_{s,k}{\left|h_{CSN-CRN,k}\right|}^{2}}{\lambda p_{p,k}{\left|h_{PT-CRN,k}\right|}^{2}+\lambda \sigma_{1,k}^{2}+\sigma_{2,k}^{2}}\right) \end{array}$$

where $\sigma_{2,k}^{2}$ denotes the noise power caused by the signal conversion.

In the second phase, CRN divides the subcarriers into two disjoint sets $G_{1}$ and $G_{2}$, where $G_{1}$ is used to transmit PT signal to PR and $G_{2}$ is used to transmit CSN signal to CRN. Thus, the signal received at PR and CDN can be denoted as

(6) $$\begin{array}{ccc} y_{k}^{PR} & = & \sum_{k^{\prime }\in G_{1}}\left(\sqrt{p_{rp,k^{\prime }}}x_{p,k^{\prime }}h_{CRN-PR,k^{\prime }}+n_{3,k^{\prime }}\right) \end{array}$$

(7) $$\begin{array}{ccc} y_{k}^{CDN} & = & \sum_{k^{\in }G_{2}}\left(\sqrt{p_{rc,k^{\prime }}}x_{s,k^{\prime }}h_{CRN-CDN,k^{\prime }}+n_{4,k^{\prime }}\right) \end{array}$$

where $x_{p,k^{\prime }}$ and $x_{s,k^{\prime }}$ denote the signal of primary and cognitive user over subcarrier $k^{\prime }$, $p_{rp,k^{\prime }}$ and $p_{rc,k^{\prime }}$ denote the transmit power to forward primary and cognitive signal over subcarrier $k^{\prime }$, $h_{CRN-PR,k^{\prime }}$ and $h_{CRN-CDN,k}$ denote the channel coefficients of CRN→PR link and CRN→CDN link over subcarrier $k^{\prime }$, $n_{3,k^{\prime }}∼\mathrm{CN}\left(0,\sigma_{3,k}^{2}\right)$ and $n_{4,k^{\prime }}∼\mathrm{CN}\left(0,\sigma_{4,k^{\prime }}^{2}\right)$ denote the noise at PR and CDN over subcarrier $k^{\prime }$.

Then in the second phase the achievable rate of CRN→PR link and CRN→CDN link is given by

(8) $$\begin{array}{ccc} R_{CRN-PR} & = & \sum_{k^{\prime }\in G_{1}}\frac{1}{2}\ln\left(1+\frac{p_{rp,k^{\prime }}{\left|h_{CRN-PR,k^{\prime }}\right|}^{2}}{\sigma_{3,k^{\prime }}^{2}}\right) \end{array}$$

(9) $$\begin{array}{ccc} R_{CRN-CDN} & = & \sum_{k^{\prime }\in G_{2}}\frac{1}{2}\ln\left(1+\frac{p_{rc,k^{\prime }}{\left|h_{CRN-CDN,k^{\prime }}\right|}^{2}}{\sigma_{4,k^{\prime }}^{2}}\right) \end{array}$$

Thus, after two transmission phases, the achievable rate of primary and cognitive system can be denoted as

(10) $$\begin{array}{ccc} R_{P} & = & \min\left(R_{PT-CRN},R_{CRN-PR}\right) \end{array}$$

(11) $$\begin{array}{ccc} R_{C} & = & \min\left(R_{CSN-CRN},R_{CRN-CDN}\right) \end{array}$$

## 4. Problem Formulation and Optimal Solutions

### 4.1. Problem Formulation

The target is to maximize the achievable rate of the cognitive system while guaranteeing the primary system achieves its target transmission rate under the transmit power constraints, through optimizing the power splitting ratio λ, power allocation $P=\{p_{rp,k^{\prime }},p_{rc,k^{\prime }}\}$ and subcarriers allocation $G=\{G_{1},G_{2}\}$. This optimization problem can be given as:

(12) $$\max_{\left\{\lambda ,P,G\right\}}R_{C}$$

subject to:

(13) $$\begin{array}{c} \left\{\begin{array}{c} \sum_{k\in K}\frac{1}{2}\ln\left(1+\frac{\lambda p_{p,k}{\left|h_{PT-CRN,k}\right|}^{2}}{\lambda p_{s,k}{\left|h_{CSN-CRN,k}\right|}^{2}+\lambda \sigma_{1,k}^{2}+\sigma_{2,k}^{2}}\right)\geq R_{T}\\ \sum_{k^{\prime }\in G_{1}}\frac{1}{2}\ln\left(1+\frac{p_{rp,k^{\prime }}{\left|h_{CRN-PR,k^{\prime }}\right|}^{2}}{\sigma_{3,k^{\prime }}^{2}}\right)\geq R_{T}\\ \sum_{k^{\prime }\in G_{1}}p_{rp,k^{\prime }}+\sum_{k^{\prime }\in G_{2}}p_{rc,k^{\prime }}\leq \sum_{k\in K}\frac{1}{2}\left(1-\lambda \right)\eta \left(p_{p,k}{\left|h_{PT-CRN,k}\right|}^{2}+p_{s,k}{\left|h_{CSN-CRN,k}\right|}^{2}+\sigma_{1,k}^{2}\right)\\ 0<p_{rp,k^{\prime }}\leq \bar{p_{r}}\\ 0<p_{rc,k^{\prime }}\leq \bar{p_{r}}\\ 0\leq \lambda \leq 1 \end{array}\right. \end{array}$$

where $R_{T}$ denotes the primary target transmission rate, $\bar{p_{r}}$ denotes the maximum transmit power over subcarrier $k^{\prime }$, the third constraint denotes that the power used by CRN in the second phase to forward primary and cognitive signals should be smaller than the energy harvested in the first phase.

### 4.2. Optimal Solutions

In Equation (13), we can find that λ is a positive value between 0 and 1, with which it is easy to obtain the optimal value. Thus, the optimal power splitting ratio $\lambda^{*}$ can be obtained through binary search. Thus, the optimization problem can be rewritten as

(14) $$\max_{\left\{P,G\right\}}R_{C}$$

subject to:

(15) $$\begin{array}{c} \left\{\begin{array}{c} \sum_{k\in K}\frac{1}{2}\ln\left(1+\frac{\lambda p_{p,k}{\left|h_{PT-CRN,k}\right|}^{2}}{\lambda p_{s,k}{\left|h_{CSN-CRN,k}\right|}^{2}+\lambda \sigma_{1,k}^{2}+\sigma_{2,k}^{2}}\right)\geq R_{T}\\ \sum_{k^{\prime }\in G_{1}}\frac{1}{2}\ln\left(1+\frac{p_{rp,k^{\prime }}{\left|h_{CRN-PR,k^{\prime }}\right|}^{2}}{\sigma_{3,k^{\prime }}^{2}}\right)\geq R_{T}\\ \sum_{k^{\prime }\in G_{1}}p_{rp,k^{\prime }}+\sum_{k^{\prime }\in G_{2}}p_{rc,k^{\prime }}\leq \sum_{k\in K}\frac{1}{2}\left(1-\lambda \right)\eta \left(p_{p,k}{\left|h_{PT-CRN,k}\right|}^{2}+p_{s,k}{\left|h_{CSN-CRN,k}\right|}^{2}+\sigma_{1,k}^{2}\right)\\ 0<p_{rp,k^{\prime }}\leq \bar{p_{r}}\\ 0<p_{rc,k^{\prime }}\leq \bar{p_{r}} \end{array}\right. \end{array}$$

Due to the constraints in Equation (15), we can see the joint optimization problem in Equation (12) is non-convex. However, when the number of subcarriers is large enough as well as when the time sharing condition is met, the optimal power allocation P and subcarriers allocation G can be obtained by using the dual decomposition method [31] with the following two steps.

### 4.3. Optimizing Dual Variables

We can obtain the Lagrange dual function of the problem in Equation (13) as

(16) $$g\left(\alpha \right)=\max_{\left\{P,G\right\}}L\left(P,G\right)$$

where

(17) $$\begin{array}{ccc} L(P,G) & = & \alpha_{1}\left(R_{CSN-CRN}-R_{C}\right)+\alpha_{2}\left(R_{CRN-CDN}-R_{C}\right)+R_{C}\\ & + & \alpha_{3}\left(\sum_{k\in K}\frac{1}{2}\left(1\;\;-\;\;\lambda \right)\eta \left(p_{p,k}{\left|h_{PT-CRN,k}\right|}^{2}\;\;+\;\;p_{s,k}{\left|h_{CSN-CRN,k}\right|}^{2}\;\;+\;\;\sigma_{1,k}^{2}\right)\;\;-\;\;\sum_{k^{\prime }\in G_{1}}p_{rp,k^{\prime }}\;\;-\;\;\sum_{k^{\prime }\in G_{2}}p_{rc,k^{\prime }}\right)\\ & + & \alpha_{4}\left(\sum_{k\in K}\frac{1}{2}\ln\left(1+\frac{\lambda p_{p,k}{\left|h_{PT-CRN,k}\right|}^{2}}{\lambda p_{s,k}{\left|h_{CSN-CRN,k}\right|}^{2}+\lambda \sigma_{1,k}^{2}+\sigma_{2,k}^{2}}\right)-R_{T}\right)\\ & + & \alpha_{5}\left(\sum_{k^{\prime }\in G_{1}}\frac{1}{2}\ln\left(1+\frac{p_{rp,k^{\prime }}{\left|h_{CRN-PR,k^{\prime }}\right|}^{2}}{\sigma_{3,k^{\prime }}^{2}}\right)-R_{T}\right) \end{array}$$

where $\alpha =\left(\alpha_{1},\alpha_{2},\alpha_{3},\alpha_{4},\alpha_{5}\right)$ is the vector of the optimal dual variables. The subgradient-based methods can be used to obtain the optimal value of α. To make sure that the dual function is bounded, we have $1-\alpha_{1}-\alpha_{2}=0$. Thus, after some manipulation, we can rewrite Equation (17) as

(18) $$\begin{array}{ccc} L(P,G) & = & \frac{\alpha_{1}}{2}\sum_{k\in K}\ln\left(1+\frac{\lambda p_{s,k}{\left|h_{CSN-CRN,k}\right|}^{2}}{\lambda p_{p,k}{\left|h_{PT-CRN,k}\right|}^{2}+\lambda \sigma_{1,k}^{2}+\sigma_{2,k}^{2}}\right)\\ & + & \frac{1-\alpha_{1}}{2}\sum_{k^{\prime }\in G_{2}}\ln\left(1+\frac{p_{rc,k^{\prime }}{\left|h_{CRN-CDN,k^{\prime }}\right|}^{2}}{\sigma_{4,k^{\prime }}^{2}}\right)\\ & + & \alpha_{3}\left(\sum_{k\in K}\frac{1}{2}\left(1\;\;-\;\;\lambda \right)\eta \left(p_{p,k}{\left|h_{PT-CRN,k}\right|}^{2}\;\;+\;\;p_{s,k}{\left|h_{CSN-CRN,k}\right|}^{2}\;\;+\;\;\sigma_{1,k}^{2}\right)\;\;-\;\;\sum_{k^{\prime }\in G_{1}}p_{rp,k^{\prime }}\;\;-\;\;\sum_{k^{\prime }\in G_{2}}p_{rc,k^{\prime }}\right)\\ & + & \frac{\alpha_{4}}{2}\left(\sum_{k\in K}\ln\left(1+\frac{\lambda p_{p,k}{\left|h_{PT-CRN,k}\right|}^{2}}{\lambda p_{s,k}{\left|h_{CSN-CRN,k}\right|}^{2}+\lambda \sigma_{1,k}^{2}+\sigma_{2,k}^{2}}\right)-2R_{T}\right)\\ & + & \frac{\alpha_{5}}{2}\left(\sum_{k^{\prime }\in G_{1}}\ln\left(1+\frac{p_{rp,k^{\prime }}{\left|h_{CRN-PR,k^{\prime }}\right|}^{2}}{\sigma_{3,k^{\prime }}^{2}}\right)-2R_{T}\right) \end{array}$$

### 4.4. Optimizing P and G with Given Dual Variables

The optimal value of P and G are obtained through the following two procedures with a given α. Firstly, the optimal P is obtained with a fixed G; secondly, the optimal G is obtained.

**1. Obtaining optimal P for fixed G**

Taking the partial derivatives of $L\left(P,G\right)$ with $p_{rp,k^{\prime }}$ and $p_{rc,k^{\prime }}$, respectively, we can obtain

(19) $$\begin{array}{ccc} \frac{\partial L}{\partial p_{rp,k^{\prime }}} & = & \frac{\alpha_{5}{\left|h_{CRN-PR,k^{\prime }}\right|}^{2}}{2(\sigma_{3,k^{\prime }}^{2}+p_{rp,k^{\prime }}{\left|h_{CRN-PR,k^{\prime }}\right|}^{2})}-\alpha_{3} \end{array}$$

(20) $$\begin{array}{ccc} \frac{\partial L}{\partial p_{rc,k^{\prime }}} & = & \frac{\left(1-\alpha_{1}\right){\left|h_{CRN-CDN,k^{\prime }}\right|}^{2}}{2(\sigma_{4,k^{\prime }}^{2}+p_{rc,k^{\prime }}{\left|h_{CRN-CDN,k^{\prime }}\right|}^{2})}-\alpha_{3} \end{array}$$

The optimal $p_{rp,k^{\prime }}$ and $p_{rc,k^{\prime }}$ can be obtained by using the KKT conditions, when the partial derivatives are equal to zero. Thus, we can obtain the optimal power allocation as

(21) $$\begin{array}{ccc} p_{rp,k^{\prime }}^{*} & = & {\left[\frac{\alpha_{5}}{2\alpha_{3}}-\frac{\sigma_{3,k^{\prime }}^{2}}{{\left|h_{CRN-PR,k^{\prime }}\right|}^{2}}\right]}_{0}^{{\bar{p}}_{r}} \end{array}$$

(22) $$\begin{array}{ccc} p_{rc,k^{\prime }}^{*} & = & {\left[\frac{\left(1-\alpha_{1}\right)}{2\alpha_{3}}-\frac{\sigma_{4,k^{\prime }}^{2}}{{\left|h_{CRN-CDN,k^{\prime }}\right|}^{2}}\right]}_{0}^{{\bar{p}}_{r}} \end{array}$$

**2. Obtaining optimal G**

Substituting Equations (20) and (21) into Equation (16) and after some manipulation, L(P,G) can be rewritten as

(23) $$\begin{array}{ccc} L(P,G) & = & \sum_{k^{\prime }\in G_{1}}F_{k^{\prime }}+\sum_{k^{\prime }\in K}\left(\frac{1-\alpha_{1}}{2}\ln\left(1+\frac{p_{rc,k^{\prime }}^{*}{\left|h_{CRN-CDN,k^{\prime }}\right|}^{2}}{\sigma_{4,k^{\prime }}^{2}}\right)-\alpha_{3}p_{rc,k^{\prime }}\right)-\frac{\alpha_{4}+\alpha_{5}}{2}R_{T}\\ & + & \sum_{k\in K}\left(\frac{\alpha_{1}}{2}\ln\left(1+\frac{\lambda p_{s,k}{\left|h_{CSN-CRN,k}\right|}^{2}}{\lambda p_{p,k}{\left|h_{PT-CRN,k}\right|}^{2}+\lambda \sigma_{1,k}^{2}+\sigma_{2,k}^{2}}\right)+\alpha_{3}\left(1-\lambda \right)\eta M_{k}\right) \end{array}$$

where $F_{k^{\prime }}\;\;=\;\;\frac{\alpha_{5}}{2}\ln\left(1\;\;+\;\;\frac{p_{rp,k^{\prime }}^{*}{\left|h_{CRN-PR,k^{\prime }}\right|}^{2}}{\sigma_{3,k^{\prime }}^{2}}\right)\;\;-\;\;\frac{1\;\;-\;\;\alpha_{1}}{2}ln\left(1\;\;+\;\;\frac{p_{rc,k^{\prime }}^{*}{\left|h_{CRN-CDN,k^{\prime }}\right|}^{2}}{\sigma_{4,k^{\prime }}^{2}}\right)\;\;+\;\;\alpha_{3}\left(p_{rc,k^{\prime }}^{*}\;\;-\;\;p_{rp,k^{\prime }}^{*}\right)$ and $M_{k}=p_{p,k}{\left|h_{PT-CRN,k}\right|}^{2}+p_{s,k}{\left|h_{CSN-CRN,k}\right|}^{2}+\sigma_{1,k}^{2}$.

In the right-hand side of Equation (22), we can find that only the first term $F_{k^{\prime }}$ is related to $G_{1}$. Thus, we can obtain the optimal $G_{1}$ through solving $F_{k^{\prime }}$

(24) $$\begin{array}{c} G_{1}^{*}=\arg\max_{G_{1}}\sum_{k\in G_{1}}F_{k^{\prime *}}. \end{array}$$

Then, all the subcarrier *k*, which have a positive $F_{k^{\prime }}$ form the optimal subcarrier allocation $G_{1}^{*}$. Then, the optimal $G_{2}^{*}$ can be obtained as

(25) $$\begin{array}{c} G_{2}^{*}=K-G_{1}^{*} \end{array}$$

**Figure 2:**
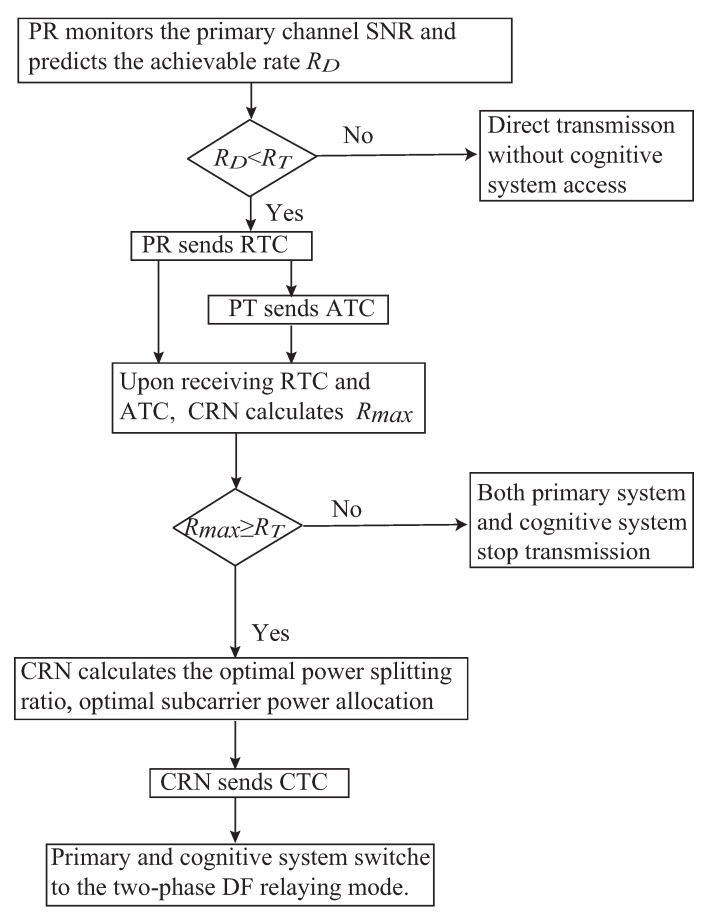
Flowchart of the proposed opportunistic spectrum sharing protocol.

With the above two steps, the optimal primal variables P and G can be obtained with given dual variables. Then the joint optimization problem in Equation (13) can be finally solved through updating the dual variables. Based on the above discussions, a detailed flowchart of the proposed opportunistic spectrum sharing protocol is illustrated in Figure 2.

## 5. Simulation Results and Discussion

We will show the simulation results to illustrate the performance of our proposed protocol in this section. Subcarriers number is set to be 32. The transmit power of CSN $P_{c}=1$ W. The energy conversion efficiency η=1. The distance of PT→CRN link, CSN→CRN link, CRN→PR link and CRN→CDN link is set to be 1.5 m.

**Figure 3:**
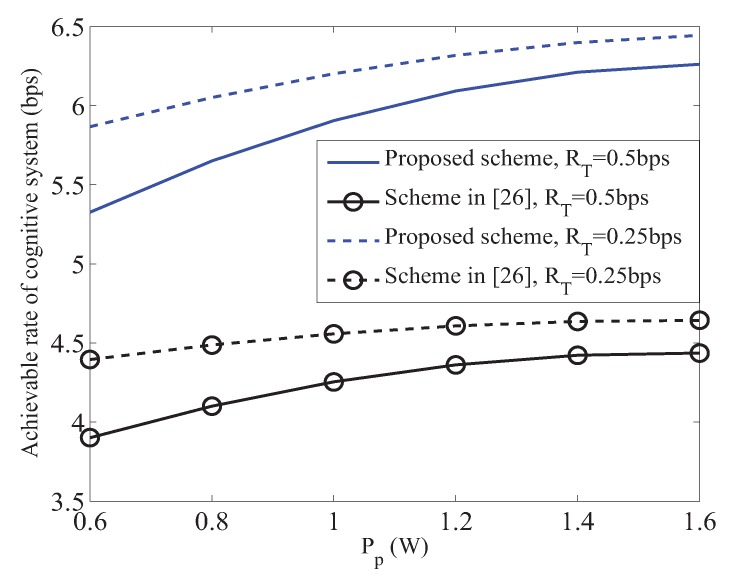
Achievable rate of cognitive system versus $P_{p}$.

**Figure 4:**
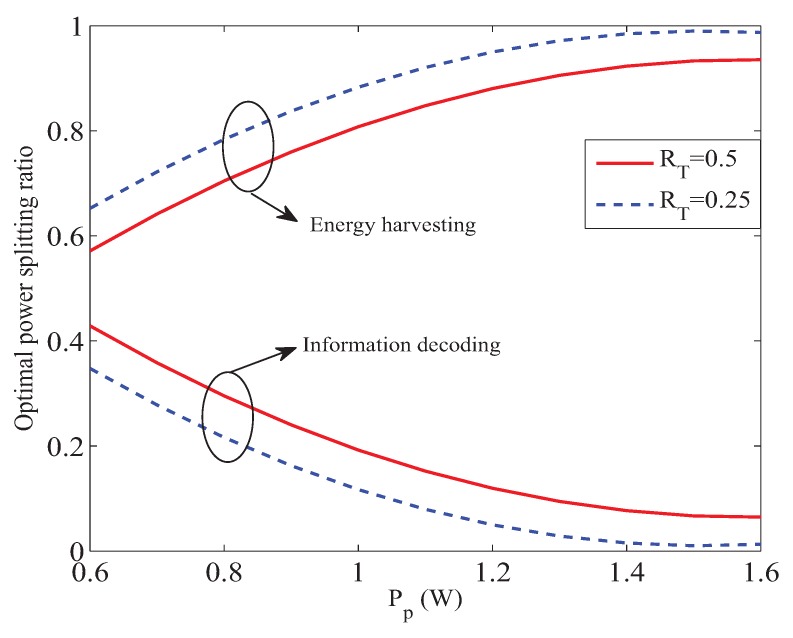
Optimal power splitting ratio in the first phase.

Figure 3 shows the achievable rate of cognitive system with different primary target transmission rate versus the transmit power of primary system. In Figure 3, we can find that our proposed scheme outperforms the scheme proposed in [26]. Because, in the scheme proposed in [26], CRN uses the same bandwidth to forward the signals of the primary and cognitive systems, they will cause interferences to each other. Thus, leading to poor performance. We can also observe from Figure 3, when $P_{p}$ increases, the achievable rate of cognitive system becomes larger. Because there is a larger primary transmit power, the power spilt to perform information decoding will be smaller in the first phase, which can be illustrated from Figure 4. Then CRN can obtain more power to perform energy harvesting, which will benefit CRN in the second phase to have more power to forward the cognitive signal; thus leading the cognitive to achieve a larger transmission rate. In Figure 3, we can also find that the achievable rate of cognitive system will become smaller when the primary target transmission rate becomes larger, which is due to more power being used for forwarding the primary signal when $R_{T}$ becomes larger.

Figure 4 shows optimal power splitting ratio in the first phase versus $P_{p}$, when the primary target transmission rate $R_{T}$ are equal to 0.5 bps and 0.25 bps, respectively. In Figure 4, we can see that when $P_{p}$ becomes larger, CRN will obtain more power from energy harvesting to forward the primary and cognitive signals in the second phase, which results in larger transmit rate of the cognitive system with the fixed primary target transmission rate. We can also see from Figure 4 that with fixed primary transmit power, more power will be split to harvest energy with smaller primary target transmission rate. With larger primary target transmission rate, more power will be needed to be allocated for information decoding, which leads to less power left for the energy harvesting.

**Figure 5:**
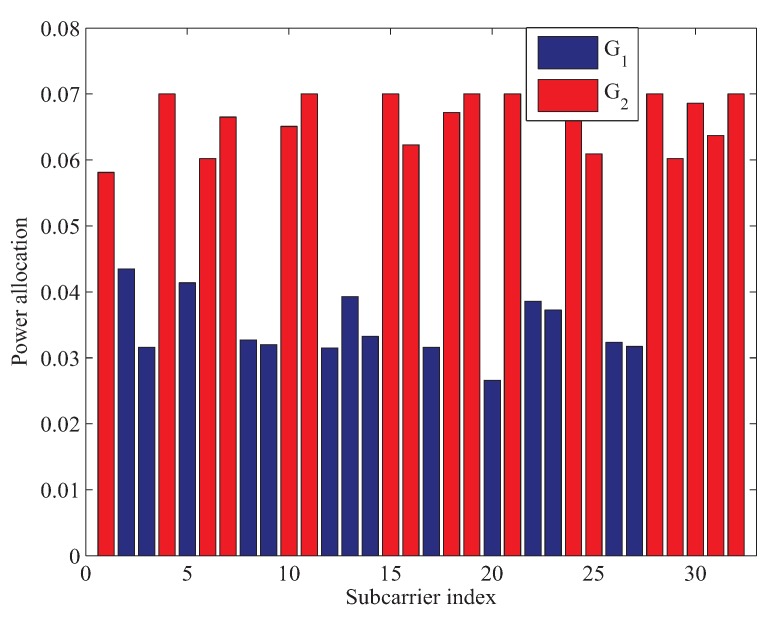
Power and subcarrier allocation in the second phase.

Figure 5 shows the optimal power and subcarrier allocation in the second phase when $P_{p}=1$ W and $R_{T}=0.5$ bps. In Figure 5, we can find that CRN utilizes disjoint subcarriers $G_{1}$ and $G_{2}$ to forward the signals of primary and second systems. Therefore, neither the primary system nor the second system is disturbed. We can also observe from Figure 5 that more subcarriers and power are allocated to forward the signal of the cognitive system, which is because only a small fraction of subcarriers and power are needed to forward the primary system to achieve with a relatively small target transmission rate, which is illustrated in Figure 3.

## 6. Conclusions

We propose a cooperative spectrum sharing protocol with two transmission phases for OFDM relaying networks with wireless energy harvesting in this paper. In the proposed protocol, CRN utilizes disjoint subcarriers to help forward the signals of primary and cognitive systems. Thus, both primary and cognitive systems will not interfere with each other to deteriorate the performance. Specifically, a fraction of the power received at CRN from the PT and CSN transmission will be split to harvest energy, and the remaining fraction of power is utilized for information decoding. CRN uses disjoint subcarriers $G_{1}$ and $G_{2}$ to forward primary and cognitive signals with the harvested energy in the first phase. The joint optimization problem, including optimal power splitting ratio, power allocation and subcarriers allocation, is studied to maximize the cognitive transmission rate with the constraint of primary target transmission rate. Numerical and simulation results are provided to give useful insights into the proposed cooperative spectrum sharing protocol and to emphasize the impact of various system parameters.
